# Analysis of cutting ballon versus plain balloon dilatation after electrified wire in situ fenestration

**DOI:** 10.1016/j.jvssci.2026.100417

**Published:** 2026-03-12

**Authors:** Marcello Silvano, Teodora Ormandzhieva, Eberhard Grambow, Florian Elger

**Affiliations:** a Department of Cardiac, Thoracic and Vascular Surgery, University Medical Centre Göttingen, Göttingen, Germany; b Division of Vascular and Endovascular Surgery, Department of Cardiac, Thoracic, Vascular Sciences and Public Health, University of Padua, Padua, Italy; c Department of General, Visceral, Thoracic, Vascular and Transplantation Surgery, University Medical Centre Rostock, Rostock, Germany

**Keywords:** Aorta, In situ fenestration, Endograft, Aneurysm, Electrified wire, Cutting balloon

## Abstract

**Objective:**

Electrified wire in situ fenestration (EW-ISF) has been recently described both in vivo and in vitro as a feasible alternative to laser ISF to address complex aortic pathologies in emergency situations. However, no data are currently available regarding the preferred dilatation method after puncture. The aim of this paper was to compare two established schemes of fenestration dilation after an EW-ISF setting.

**Methods:**

A total of 72 EW-ISFs were performed in 4 commercially available endografts: Zenith Alpha (n = 20), RelayPro (n = 20), Endurant IIs (n = 20), and Valiant Captivia (n = 12). In group A, fenestrations were sequentially dilatated with a 2-mm and a 6-mm plain balloon dilatation (PBD). In group B, fenestrations were dilatated with a 2-mm PBD, a 4-mm cutting balloon, and a 6-mm PBD. Postdilatation fenestration morphological features were analyzed, and diameters and surface were measured after dilatation and after 24 hours to assess the relative fabric elastic recoil. For each graft, in both group one bridging stent (BS) was implanted and its caliber measured with intravascular ultrasound.

**Results:**

In both the Zenith and the Endurant, group B showed more tearing (*P* = .302 and *P* < .001 respectively), less bulging (*P* = .039 and *P* < .001), and more frequent slit-like fenestration morphology (*P* = .015 and *P* = .001). The Valiant graft displayed major (>0.5 cm long) tearing in group B. Fenestration dimensions and recoil momentum were comparable between the two groups in all grafts, but the Endurant, where group B fenestrations had significantly higher fenestration area (6.5 mm^2^ vs 2.3 mm^2^; *P* < .001). BS caliber was comparable in both groups in the RelayPro and Zenith Alpha, but a significant stenosis was assessed in group A in the Endurant IIs.

**Conclusions:**

Both cutting balloon dilatation and PBD are feasible and comparable after EW-ISF in the in the RelayPro and the Zenith grafts. Cutting balloon provides more ideal fenestration dimensions in the Endurant IIs and might be preferred. It determines excessive damages in the monofilament graft and should, therefore, be avoided.

**Clinical Relevance:**

Electrified wire in situ fenestration is an emerging, low-cost alternative for urgent aortic branch revascularization, but optimal postfenestration dilation remains undefined. This study provides practical guidance on balloon selection according to graft fabric. Cutting balloons may improve fenestration enlargement and reduce the risk of bridging stent stenosis in multifilament grafts such as Endurant IIs, while causing excessive damage in monofilament devices. Tailoring dilation strategy to graft type may enhance safety, durability, and procedural success in emergency endovascular aortic repair.

**Article Highlights**

• **Type of Research:** In vitro study.
• **Key Findings:** Plain balloon dilation of electrified wire in situ fenestration yielded less tearing than cutting balloon dilation, especially in monofilament grafts, where major tearing were seen. The achieved fenestration dimension in the Endurant graft was satisfactory only after cutting ballon dilation, determining instead significant bridging stent grafts stenosis after plain balloon dilatation.
• **Take Home Message:** Both cutting balloon and plain balloon may be feasible dilation methods after electrified wire in situ fenestration in multifilament grafts, such as the RelayPro and the Zenith Alpha. Cutting balloon should be preferred in the Endurant IIs, but avoided in the Valiant Captivia.

The standard of care for complex abdominal and thoracoabdominal aneurysms is represented by endovascular management with fenestrated/branched custom-made or off-the-shelf stent grafts. However, emergency situations and anatomical complexity may preclude these alternatives. ISF of aortic stent grafts has emerged as a valid alternative in urgent endovascular treatment, allowing maintenance of target vessel patency both in abdominal and thoracoabdominal scenarios.^1^ Traditionally, ISF has been frequently performed using dedicated laser systems (L-ISF),^2^, ^3^, ^4^ but such devices are costly and not always available. Recently, a new energy-based ISF method using an electrified standard guidewire (EW-ISF) has been described both in vitro^5^^,^^6^ and in vivo^7^^,^^8^ as a feasible off-the-shelf solution when L-ISF is not applicable or available.

Even if limited evidence regarding EW-ISF exists, the technique seems reproducible, associated with a low puncture failure rate and capable of obtaining high-quality fenestrations in vitro.^6^ However, even if the puncture technique is well-established, details regarding the best method to obtain the fenestration dilation, before bridging stent deployment, are still missing. Moreover, no robust comparative data are currently available regarding the performance of the technique in different available abdominal and thoracic stent grafts. Some authors used a cutting balloon dilatation (CBD) after ISF with small fenestration devices^9^^,^^10^ to enhance the fenestration dimension and ease the bridging stent (BS) accommodation in real-life scenarios. However, other studies suggested that such an approach may increase the risk of graft tearing in the long term,^11^ supporting the use of standalone plain balloon dilatation (PBD),^12^, ^13^, ^14^, ^15^ possibly using multiple balloons in series to approach small initial ISF.^16^

The aim of this study was to compare two well-established predilation methods, using PBD as a standalone method or combined with CBD after EW-ISF in different stent grafts.

## Methods

### Fenestration method and graft selection

**Fig 1:**
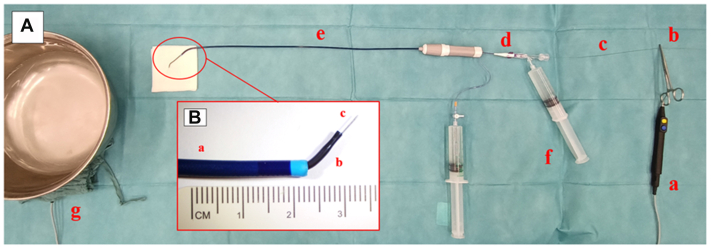
Experimental setting for electrified wire in situ fenestration (EW-ISF). **(A) (a)** Monopolar cautery. **(b)** De-insulated portion of the guidewire. **(c)** The 0.018′ guidewire body. **(d)** Supporting 5F catheter. **(e)** The 7F steerable sheath. **(f)** Flushing with 5% glucose. **(g)** Saline bath container and indifferent electrode. Particular of electrified wire setting. **(B) (a)** The 7F steerable sheath. **(b)** Supporting 5F catheter. **(c)** The 0.018 guidewire tip.

All EW-ISF were performed according to the same method previously well-described in the literature.^5^^,^^6^ The hydrophilic coating was removed from the proximal end of 0.018″ Astato 30 guidewire (Asahi Intecc) via a scalpel blade over approximately 2 cm in length to obtain a proper de-insulation. The samples were submerged in a saline bath at 37°C in a metal container. The indifferent electrode pad was connected to the bottom of the saline bath container. The wire was positioned perpendicularly to the graft surface and supported with a vertebral catheter (Impress Legato 4F angiographic catheter, Merit Medical Systems) inside a standard 6F sheath. The sheath was constantly flushed with a 5% glucose solution. The graft puncture was obtained by gently advancing the wire thorough the fabric while applying an electric burst to the wire proximal end with a monopolar cautery connected to the electrosurgical unit (VIO 300D monopolar cautery, Erbe Elektromedizin). The device was set in cut mode at 180 W power (Fig 1).

Fenestrations were separately performed in four polyester grafts: Zenith Alpha (n = 20) (Cook Medical), RelayPro (n = 20) (Terumo Aortic), and Endurant IIs (n = 20) and Valiant Captivia (n = 12) (both Medtronic Vascular).

### Dilatation protocol

Two different dilatation schemes, already established in the literature were used and systematically compared in each graft. Group A included two sequential PBD dilations were performed with a 2 × 20 mm noncompliant balloon (Sterling, Boston Scientific) and a 6 × 20 mm noncompliant balloon (Admiral Xtreme, Medtronic), both inflated at nominal pressure for 20 seconds. The number of fenestrations was as follows: Zenith Alpha (n = 10), RelayPro (n = 10), Endurant IIs (n = 10), and Valiant Captivia (n = 6).

Group B comprised fenestrations that were dilated sequentially with a 2 × 20 noncompliant balloon (Sterling, Boston Scientific), a 4 × 15 mm cutting balloon (Wolverine, Boston Scientific) and a 6 × 20 mm noncompliant balloon (Admiral Xtreme, Medtronic). The number of fenestrations was as follows: Zenith Alpha (n = 10), RelayPro (n = 10), Endurant IIs (n = 10), andValiant Captivia (n = 6).

### Fenestration assessment

Microscopic images of fenestrations were obtained with a digital microscope (DM202 Max, Tomlov) and later analyzed with a dedicated software for image assessment (ImageJ 1.54h). Images were taken after puncture, immediately after the completion of the balloon dilatation process and after 24 hours.

**Fig 2:**
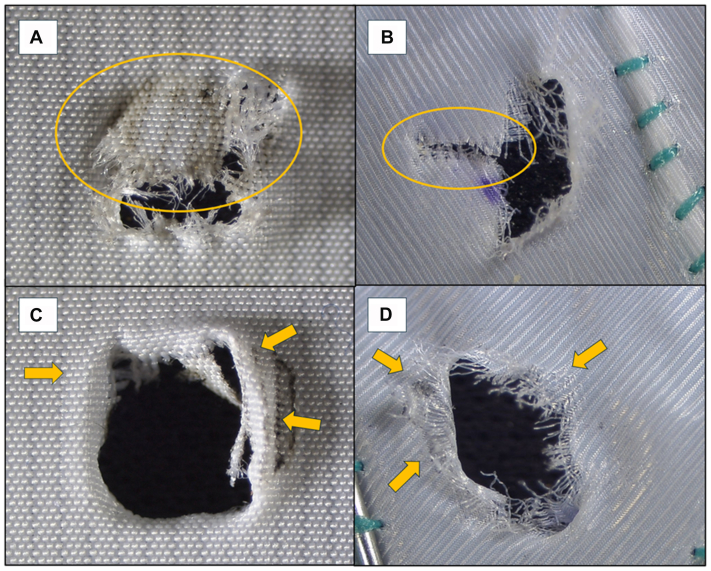
Analyzed qualitative fenestration features. Shredding after electrified wire in situ fenestration (EW-ISF) in the RelayPro **(A)**, tearing after EW-ISF in the Valiant **(B)**, bulging after EW-ISF in the RelayPro **(C)**, and fraying after EW-ISF in the Valiant **(D)**. Specific features are marked by *colored ovals* and *arrows*.

Measurements of horizontal (fabric weft direction) and vertical (fabric warp direction) diameters and fenestration surface area after dilatation and after 24 hours were recorded. Postdilatation fenestration shape and features were analyzed by two independent observers applying the following definitions as already described in the literature (Fig 2).

• Tearing: full-thickness fabric rip extending from the fenestration rim. Major tearing was defined when the tear length was >0.5 cm.
• Fraying: excessive (≥5) visible separated fabric fibers encircling the orifice.
• Shredding: graft debris partially (>1/3 of the surface) obstructing the fenestration opening.
• Bulging: >50% circumferential donut-like thickening of the fabric at the fenestration rim.

Relative elastic recoil of the fenestration was calculated as the difference of initial and 24-hour diameter and surface, expressed in percentage, in relation to the baseline value (postdilation).

The primary outcome was postdilation fenestration surface area. Secondary outcomes included fenestration diameters, shape, and morphological features (tearing, fraying, shredding, and bulging). Fenestration dimensions after 24 hours were collected as an exploratory assessment of recoil.

### Stenting and ultrasound analysis

**Fig 3:**
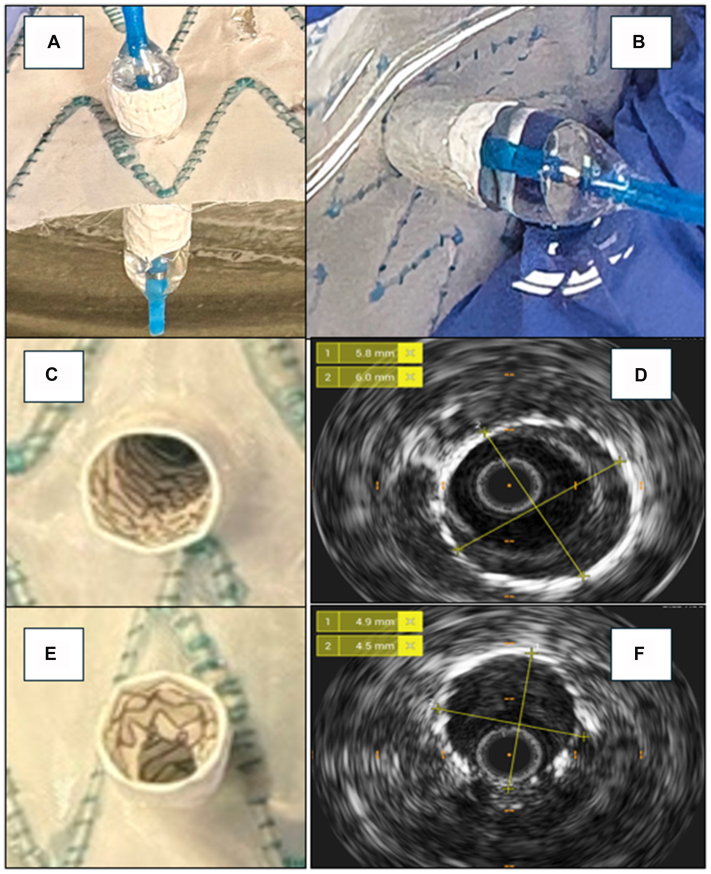
Bridging stent (BS) graft positioning. Stent graft release **(A)** and flaring **(B)**. Flared BS in group A (plain balloon only) **(C)** and relative diameter measured with intravascular ultrasound (IVUS) at the fenestration level **(D)**. Flared BS in group B (cutting balloon) **(E)** and relative diameter measured with IVUS at the fenestration level **(F)**.

For each graft in both groups, as proof of concept, one fenestration was randomly selected and stented with a 6-mm BeGraft balloon-expandible stent (Bentley InnoMed), simulating a BS in a real-life ISF application. The stent was released in a 37°C saline bath, inflated to nominal pressure, and flared with a 10 × 20 mm noncompliant balloon (Admiral Xtreme, Medtronic) at 10 atm (Fig 3).

All implanted BSs were analyzed with intravascular ultrasound after stent deployment and after flaring using a 0.018 probe (CoreM2 System, Philips Volcano), similarly to previous experiences.^17^ Diameters at the level of the fenestration were measured both after BS release and flaring, to assess the presence of a BS stenosis. The stent diameter was also measured after its release above the fenestration rim, to obtain a baseline value. BS-to-fenestration good apposition was manually assessed by light manipulation to confirm a good attachment of the BS to the graft fabric.

### Statistical analyses

The normality of distribution of the continuous variables was tested with the Shapiro-Wilk test. Given the limited numbers, continuous variables were expressed as median and interquartile range (Q3-Q1) and compared among different groups with either the independent samples *t* test (if normally distributed) or the Mann-Whitney *U* test. Categorical variables were expressed as frequencies and percentages, and the Fisher's exact test was applied for comparisons. Interobserver agreement was calculated using the intraclass correlation coefficient for absolute agreement and expressed with a 95% confidence interval. A *P* value of <.05 was considered significant. All statistical data analysis were performed with SPSS Statistics for Windows (IBM release 24).

## Results

### Fenestration qualitative assessment

After the initial fenestration, the puncture site in all grafts was consistent with previously reported findings in the literature: the opening was punctiform and surrounded by a black halo.

In both abdominal grafts, fenestration characteristics followed a similar pattern (Table I). Group A exhibited less tearing than group B (6 vs 9 for the Zenith and 0 vs 10 for the Endurant; *P* = .302 and *P* < .001, respectively) and more frequent bulging (5 vs 0 in the Zenith and 8 vs 0 in the Endurant; *P* = .039 and *P* < .001, respectively). Furthermore, fenestrations in group B more commonly displayed a slit-like morphology (6/10 cases for the Zenith and 7/10 cases for the Endurant), whereas group A was characterized by squared fenestrations in the Zenith (6/10) and round fenestrations in the Endurant (9/10) (all *P* < .005). No differences were observed in fraying or shredding.

**Table I:** Group A (sequential dilatation with noncompliant balloons) and group B (sequential dilatation with noncompliant, cutting- and noncompliant balloons) comparison regarding fenestration features and measures in two abdominal stent grafts (Endurant IIs and Zenith Alpha)

	Group A	Group B	*P* value
Zenith Alpha	(n = 10)	(n = 10)	
Fenestration features			
Tearing	6 (60)	9 (90)	.302
Fraying	8 (80)	9 (90)	1.0
Shredding	8 (80)	8 (80)	.576
Bulging	5 (50)	0	.039
Shape			
Elliptical	3 (30)	1 (10)	.576
Square	6 (60)	0	.015
Round	1 (10)	3 (30)	.576
Slit-like	0	6 (60)	.015
Postdilation measures			
Horizontal diameter, mm	2.1 (2.5-2.1)	1.9 (3.4-1.3)	.604
Vertical diameter, mm	3.7 (4.0-3.5)	3.1 (5.5-1.5)	.842
Surface, mm^2^	6.9 (7.8-5.8)	5.8 (7.6-4.8)	.400
24-Hour assessment			
Horizontal recoil, %	11.0 (14.3-5.9)	6.9 (11.4-1.7)	.115
Vertical recoil, %	3.8 (25.0-0.0)	9.0 (18.6-3.1)	.447
Surface recoil, %	7.1 (11.1-0.7)	7.1 (20.4-3.5)	.193
Surface, mm^2^	6.5 (7.8-5.5)	5.7 (6.9-4.0)	.315
Endurant IIs	(n = 10)	(n = 10)	
Fenestration features			
Tearing	0	10 (100)	<.001
Fraying	10 (100)	10 (100)	-
Shredding	6 (60)	7 (70)	.639
Bulging	8 (80)	0	<.001
Shape			
Elliptical	1 (1)	0	.305
Square	0	3 (30)	.060
Round	9 (90)	0	<.001
Slit-like	0	7 (70)	.001
Postdilation measures			
Horizontal diameter, mm	1.7 (2.0-1.6)	2.4 (3.1-1.7)	.031
Vertical diameter, mm	2.1 (2.4-1.7)	3.5 (4.5-2.6)	.003
Surface, mm^2^	2.3 (2.7-2.1)	6.5 (7.8-5.5)	<.001
24-Hour assessment			
Horizontal reoil, %	16.5 (21.7-6.2)	9.4 (23.9-4.4)	.925
Vertical recoil, %	13.4 (20.5-6.2)	12.2 (15.9-7.8)	.912
Surface recoil, %	5.5 (11.2-2.4)	10.2 (13.3-5.8)	.340
Surface, mm^2^	2.3 (2.5-2.0)	5.9 (7.2-5.1)	<.001

Values are number (%) or median (interquartile range).

Conversely, the thoracic grafts demonstrated a different behavior. In the RelayPro, group A exhibited more frequent bulging (6 vs 0; *P* = .015) but less frequent shredding (5 vs 10; *P* = .039), whereas tearing was comparable between groups. Both groups showed considerable variability in fenestration shape without significant differences (Table II).

**Table II:** Group A (sequential dilatation with noncompliant balloons) and group B (sequential dilatation with noncompliant, cutting- and noncompliant balloons) comparison regarding fenestration features and measures in two thoracic stent grafts (RelayPro and Valiant Captivia)

	Group A	Group B	*P* value
RelayPro	(n = 10)	(n = 10)	
Fenestration features			
Tearing	8 (80)	10 (100)	.456
Fraying	9 (90)	10 (100)	1.0
Shredding	5 (50)	10 (100)	.039
Bulging	6 (60)	0	.015
Shape			
Elliptical	3 (30)	1 (10)	.576
Square	1 (10)	0	1.0
Round	2 (20)	4 (40)	.626
Slit-like	4 (40)	5 (50)	1.0
Postdilation measures			
Horizontal diameter, mm	1.9 (2.7-1.8)	2.1 (2.5-1.6)	.929
Vertical diameter, mm	3.7 (4.2-3.5)	4.6 (5.2-3.7)	.928
Surface, mm^2^	6.0 (8.7-5.2)	7.2 (7.7-6.6)	.416
24-Hour assessment			
Horizontal recoil, %	17.1 (30.4-2.7)	10.6 (14.1-4.6)	.282
Vertical recoil, %	5.5 (32.1-0.0)	5.2 (14.6-0.5)	.705
Surface recoil, %	5.2 (11.7-3.3)	9.6 (18.9-6.6)	.099
Surface, mm^2^	5.76 (8.1-4.5)	6.4 (7.1-5.3)	.670
Valiant Captivia	(n = 6)	(n = 6)	
Fenestration features			
Tearing	2 (33.3)	5 (83.3)	.022
Fraying	6 (100)	4 (66.7)	.251
Shredding	6 (100)	6 (100)	-
Bulging	6 (100)	6 (100)	-
Shape			
Elliptical	3 (50.0)	1 (16.7)	.303
Square	3 (50.0)	1 (16.7)	.303
Round	0	1 (16.7)	.251
Slit-like	0	3 (50.0)	.026
Postdilation measures			
Horizontal diameter, mm	2.3 (2.5-1.9)	1.2 (2.2-0.8)	.055
Vertical diameter, mm	3.4 (4.4-2.3)	2.0 (3.9-1.3)	.298
Surface, mm^2^	6.9 (7.5-4.6)	2.3 (7.6-1.1)	.219
24-Hour assessment			
Horizontal reoil, %	9.0 (27.0-3.5)	1.5 (23.6-0.9)	.792
Vertical recoil, %	2.9 (26.9-1.5)	1.7 (15.3-1.2)	.662
Surface recoil, %	5.2 (8.1-2.9)	0.6 (32.6-0.4)	.662
Surface, mm^2^	6.8 (7.8-4.3)	1.7 (7.6-0.9)	.170

Values are number (%) or median (interquartile range).

**Fig 4:**
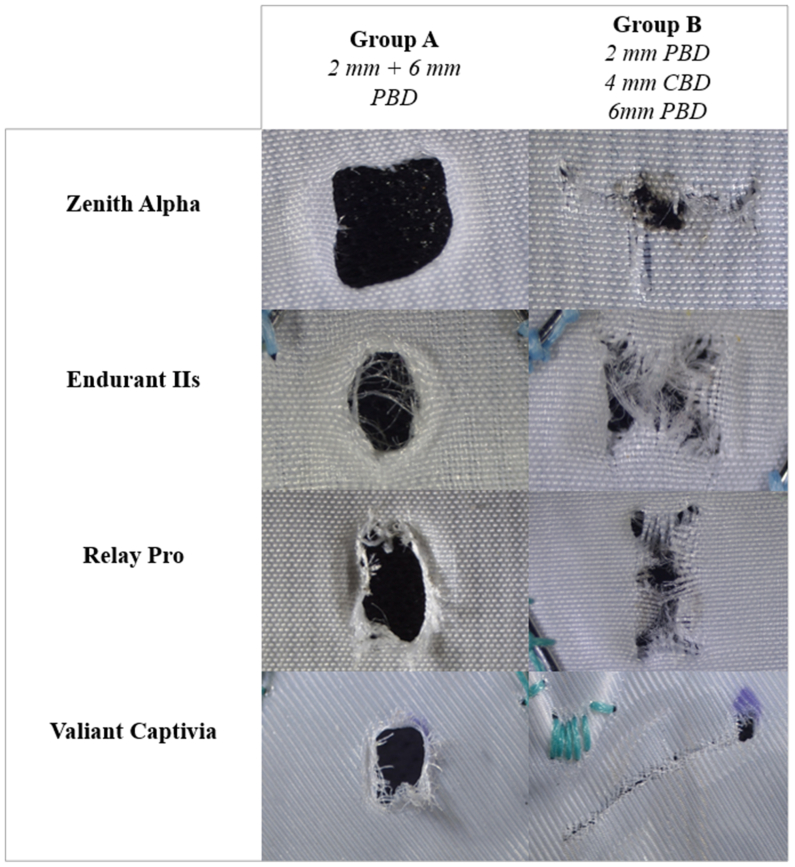
Electrified wire in situ fenestration (EW-ISF)s after dilation in different grafts. *CBD*, cutting balloon dilation; *PBD*, plain balloon dilation.

The Valiant graft was characterized by markedly greater tearing in group B (2 vs 5; *P* = .022), with some tears exceeding 1 cm and associated with a slit-like morphology (0 vs 3; *P* = .026). Tear length following the group A scheme was similar to that observed in the other grafts (Fig 4).

### Fenestration dimensions

In both the Zenith and RelayPro grafts, fenestration diameters and surface areas were generally larger in group B, although differences were not substantial. In the Valiant graft, dimensions were also broadly comparable between groups; however, fenestration area demonstrated significantly greater variability in group B compared with group A (variance of 11.58 vs 3.56), driven by substantially larger fenestrations associated with major fabric tearing. In contrast, in the Endurant graft, group B fenestrations exhibited significantly larger diameters and, in particular, surface areas (6.5 mm^2^ vs 2.3 mm^2^; *P* < .001) compared with group A. Interobserver agreement was excellent for all measured parameters (intraclass correlation coefficient >0.9) (Table III).

**Table III:** General interobserver agreement and interpretation for continuous variables

Parameter	ICC	95% CI	Interpretation
Postdilation horizontal diameter	0.991	0.988-0.995	Excellent
Postdilation vertical diameter	0.994	0.987-0.996	Excellent
Postdilation fenestration area	0.997	0.995-0.999	Excellent
Horizontal diameter after 24 hours	0.996	0.993-0.999	Excellent
Vertical diameter after 24 hours	0.994	0.991-0.997	Excellent
Area after 24 hours	0.995	0.993-0.997	Excellent

*CI,* Confidence interval; *ICC,* interclass correlation agreement.

### Fabric recoil

Fabric recoil 24 hours after the initial dilation was assessed in all samples. In both groups, the reduction in fenestration surface was moderate (<10%) across all grafts. A nonsignificant trend toward greater recoil in group B was observed in all grafts except the Valiant. An opposite trend was noted in the Valiant, possibly reflecting the absence of recoil in the presence of major tearing.

### BS expansion

**Fig 5:**
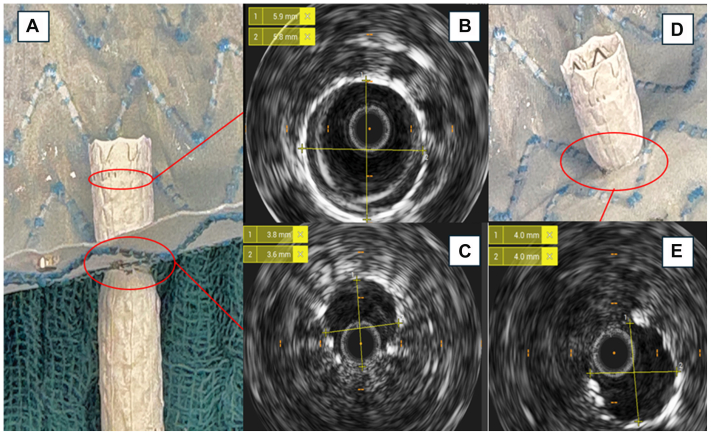
Bridging stent (BS) graft expansion measure by mean of intravascular ultrasound in the Endurant IIs graft after plain ballooning only. Implanted BS graft before flaring **(A)** and relative diameter measurements outside the fenestration **(B)** and at the fenestration level **(C)**. Stent after flaring **(D)** and diameter measurement at fenestration level **(E)**.

BS expansion was greater when CBD was used (group B) across all grafts. This difference was particularly pronounced for the Endurant II graft, where the stent diameter after flaring measured 6.3 mm in group B compared with 4.0 mm in group A, showing an important stenosis. Conversely, comparable diameters were observed for the Zenith Alpha (6.7 mm in both groups) and the RelayPro (6.7 mm in group B vs 7.1 mm in group A) (Fig 5). Overall, BS-to-fenestration apposition was satisfactory in all grafts, with the exception of the Valiant in group B, where CBD-induced tears were slightly elongated by the stenting/flaring maneuver, resulting in increased BS mobility and reduced attachment to the fenestration rim.

## Discussion

Given the relative novelty of EW-ISF as an ISF technique, this study represents the first robust investigation comparing two predilatation strategies: a PBD-only sequence and a CBD-based scheme. Both approaches have been previously described in the literature following ISF with small caliber devices.^16^^,^^18^ Several authors have reported good clinical outcomes using the CBD-based (group B) scheme after L-ISF with 0.9-mm laser fibers.^9^^,^^10^^,^^19^ However, concerns have been raised owing to the greater incidence of tearing associated with CBD, which may compromise fenestration stability and BS integrity in real-life settings.^11^ The full-PBD scheme (group A) was adopted as the reference approach after EW-ISF, based on previous in vitro evidence demonstrating satisfactory fenestration quality with limited tearing,^5^^,^^6^ and was retained in the present study to ensure methodological comparability. A recognized limitation is the lack of direct clinical validation of this specific approach. Nevertheless, comparable strategies incorporating PBD, including the sequential use of higher-caliber balloons, have been reported after L-ISF and mechanical ISF (ie, needle), with favorable outcomes.^20^^,^^21^ The decision not to include an intermediate 4-mm PBD, as implemented in the group B scheme, was intended to reflect real-world practice, in which reducing device exchanges is essential to streamline the fenestration workflow and facilitate prompt reperfusion of target vessels.

In the present study, fenestrations in group A demonstrated a quality broadly consistent with previously reported in vitro experiences. The greater incidence of fabric tearing observed in group B is likely attributable to the use of a cutting balloon, as anticipated. This mechanism may also account for the absence of bulging across all samples in three of the evaluated grafts, a finding that aligns with prior reports using a similar group B scheme after L-ISF. With respect to fenestration dimensions, both groups showed measurements generally comparable with those reported in prior on-bench EW-ISF studies for group A^5^^,^^6^ and in small caliber L-ISF experiences for group B.^18^ However, the dilatation protocol should be regarded as graft specific. The Valiant graft, characterized by a monofilament fabric architecture, appeared to be particularly susceptible to tearing propagation when exposed to cutting balloon blades, resulting in major rips that in some cases exceeded 1 cm, as reflected by the poor BS attachment to the fenestration. This observation has not been previously reported in other experiences involving cutting balloons and may be related to the thinner polyester structure of the graft and its less dense weave; however, any specific mechanistic explanation for this phenomenon remains speculative. These findings warrant caution regarding the application of the group B scheme in this graft. Based on the present results, the Valiant graft may be less suitable for ISF, potentially irrespective of the device used, although further validation would be required to substantiate this observation.

The Zenith and RelayPro grafts exhibited comparable fenestration dimensions and morphological characteristics. The use of CBD was associated with increased fabric tearing without providing a clear dimensional advantage. Accordingly, both dilatation regimens appear to be feasible, with PBD offering a more conservative impact on the fabric while achieving comparable stent expansion after delivery and flaring.

The Endurant graft demonstrated morphological features comparable with those observed in both the Zenith and RelayPro grafts. However, the use of PBD alone resulted in very small fenestrations (median area ∼2.3 mm^2^), which may be insufficient for 6-mm BSs (28.3 mm^2^ lumen), as reflected by BS stenosis and the limited stent diameters achieved.

**Fig 6:**
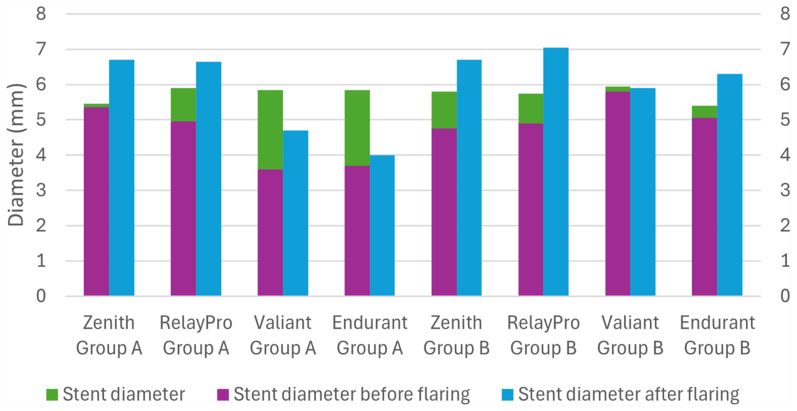
Bar chart comparing achieved stent expansion in group A (plain ballooning dilation) and B (plain and cutting ballooning).

CBD increased the fenestration area to approximately 6.5 mm^2^, which appeared to be sufficient to accommodate a 6-mm stent and resulted in a significantly greater BS expansion. These findings suggest that a CBD-based scheme may be more appropriate than PBD alone for this graft, potentially reducing the risk of in-stent stenosis (Fig 6), although the limited number of implanted stents warrants caution in interpreting this observation.

For operators wishing to avoid cutting balloons, or in emergencies when a cutting balloon is not available, escalation to high-pressure PBD with deliberately more aggressive predilatation and postdilatation/flare (within rated burst pressure and device instructions) may be considered. This strategy may partially mitigate recoil and increase the orifice diameter; however, these considerations fall outside the scope of the present study and require further investigation. In any case, L-ISF remains the preferred and clinically validated ISF technique, whereas EW-ISF currently lacks comparable clinical evidence and should, therefore, be regarded as a possible off-the-shelf alternative when costly laser devices are unavailable. A more meaningful comparison between these approaches would require long-term mechanical fatigue testing to assess fenestration durability and BS integrity, as recently performed in the comparison of L-ISF with benchmark custom-made fenestrations.^15^

Regarding fabric recoil, limited values were observed across all groups and grafts, consistent with previous reports,^6^ which suggested that disruption of fabric yarns, following energy-based methods may contribute to reduced recoil compared with mechanical fenestration strategies.^2^ Nonetheless, it remains unclear whether the recoil momentum has a meaningful impact on fenestration stability—potentially reducing the risk of BSG migration or type Ic/IIIc endoleaks^22^—or whether excessive recoil may predispose to BSG stenosis.^6^^,^^13^ The combination of small and thus tight fenestrations after EW-ISF with minimal recoil may represent a compromise in this regard.

### Limitations

This study is a pilot, on-bench exploratory study. As such, there are several limitations that are important to discuss. First, there is inherent bias in our approach because our observations were not blinded to graft type or dilation strategy. Second, the number of samples and studied grafts was small. This factor limits the usefulness of statistical results, particularly for comparing grafts with each other in head-to-head comparisons. Third, mechanical stability tests, such as long-term fatigue tests or pull-out, were not performed. Both the sample size and rigorous mechanical testing will be critical future studies to better understand clinical utility of these findings. Finally, future work using other dilation schemes may yield different results. Additionally, EW-ISF should be compared head-to-head with the current ISF benchmark (L-ISF), both in vitro and in vivo settings. This analysis may help to drive more investigation ex vivo to better understand what to expect in patients with treated with off-label ISF techniques.

## Conclusions

Both cutting balloon-based dilatation and PBD appear to be feasible as predilatation methods after EW-ISF, and no definitive preference emerged for the RelayPro and Zenith grafts. CBD may be preferred for the Endurant II graft, because it was the only approach in this study that yielded fenestrations with potentially acceptable dimensions. However, the use of a cutting balloon may be contraindicated in the Valiant Captivia graft owing to markedly greater fabric damage.

## Author Contributions

Conception and design: MS, TO, EG, FE

Analysis and interpretation: MS, TO, EG, FE

Data collection: MS, TO, EG, FE

Writing the article: MS, TO, FE

Critical revision of the article: MS, TO, EG, FE

Final approval of the article: MS, TO, EG, FE

Statistical analysis: MS, TO, FE

Obtained funding: Not applicable

Overall responsibility: MS

## Funding

None.

## Disclosures

None.
